# Development Trends and Prospects of Technology-Based Solutions for Health Challenges in Aging Over the Past 25 Years: Bibliometric Analysis

**DOI:** 10.2196/63367

**Published:** 2024-09-20

**Authors:** Lu Liu, Xiu-Ling Wang, Nuo Cheng, Fu-Min Yu, Hui-Jun Li, Yang Mu, Yonghui Yuan, Jia-Xin Dong, Yu-Dan Wu, Da-Xin Gong, Shuang Wang, Guang-Wei Zhang

**Affiliations:** 1 Department of Cardiac Surgery The First Hospital of China Medical University Shenyang China; 2 Department of Cardiology The First Hospital of China Medical University Shenyang China; 3 Department of Gynecology The First Hospital of China Medical University Shenyang China; 4 Department of Cardiovascular Surgery Guangdong Provincial People's Hospital, Guangdong Academy of Medical Sciences Southern Medical University Guangzhou China; 5 Shenyang Medical & Film Science and Technology Co. Ltd. Shenyang China; 6 Enduring Medicine Smart Innovation Research Institute Shenyang China; 7 Goodwill Information Technology Co., Ltd. Beijing China; 8 Cancer Hospital of China Medical University Liaoning Cancer Hospital & Institute Clinical Research Center for Malignant Tumor of Liaoning Province Shenyang China; 9 Department of Nursing Faculty China Medical University Shenyang China; 10 Smart Hospital Management Department The First Hospital of China Medical University Shenyang China; 11 Internet Hospital Branch Chinese Research Hospital Association Beijing China; 12 Department of General Practice The First Hospital of China Medical University Shenyang China

**Keywords:** bibliometrics, CiteSpace, VOSviewer, visualization, aging health, technological innovations, tech-based, technology-based, technology, health challenges, challenges, trends, older adults, older adult, ageing, aging, elder, elderly, older person, older people, gerontology, geriatric, geriatrics, remote, remote monitoring, monitoring, surveillance, artificial intelligence, AI, AI-driven, innovation, innovations, health management, telemedicine, remote care

## Abstract

**Background:**

As the global population ages, we witness a broad scientific and technological revolution tailored to meet the health challenges of older adults. Over the past 25 years, technological innovations, ranging from advanced medical devices to user-friendly mobile apps, are transforming the way we address these challenges, offering new avenues to enhance the quality of life and well-being of the aging demographic.

**Objective:**

This study aimed to systematically review the development trends in technology for managing and caring for the health of older adults over the past 25 years and to project future development prospects.

**Methods:**

We conducted a comprehensive bibliometric analysis of literatures related to technology-based solutions for health challenges in aging, published up to March 18, 2024. The search was performed using the Web of Science Core Collection, covering a span from 1999 to 2024. Our search strategy was designed to capture a broad spectrum of terms associated with aging, health challenges specific to older adults, and technological interventions.

**Results:**

A total of 1133 publications were found in the Web of Science Core Collection. The publication trend over these 25 years showed a gradual but fluctuating increase. The United States was the most productive country and participated in international collaboration most frequently. The predominant keywords identified through this analysis included “dementia,” “telemedicine,” “older-adults,” “telehealth,” and “care.” The keywords with citation bursts included “telemedicine” and “digital health.”

**Conclusions:**

The scientific and technological revolution has significantly improved older adult health management, particularly in chronic disease monitoring, mobility, and social connectivity. The momentum for innovation continues to build, with future research likely to focus on predictive analytics and personalized health care solutions, further enhancing older adults’ independence and quality of life.

## Introduction

As the global population aging trend continues to intensify, the health issues of older adults have increasingly become the focus of global attention [1]. As a pivotal force in the scientific and technological revolution addressing global aging, a diverse array of technologies is reshaping how we meet the health challenges of older adults [2]. By harnessing technological innovations, ranging from cutting-edge medical devices to user-friendly mobile apps, novel avenues to enhance the quality of life and well-being of the aging demographic are unveiled [3]. Among these advancements are remote health monitoring systems capable of real-time tracking of vital signs, assistive technologies fostering independence in daily activities, and the integration of artificial intelligence (AI) and data analytics, revolutionizing health care delivery and enabling tailored approaches to personalized medicine for older adults [4-6]. Moreover, the indispensability of telemedicine and virtual health care solutions has been underscored, particularly in the aftermath of the COVID-19 pandemic, highlighting their crucial role in delivering remote care and consultations [7,8]. These technologies empower older adults to access health care services from the convenience of their homes, curtailing the necessity for physical clinic visits and mitigating exposure to infectious diseases [9]. When an accident occurs, older adults can seek emergency help remotely, and nurses can observe and understand the situation through videos, providing targeted medical assistance [10]. In essence, technology stands as a transformative catalyst in confronting the health complexities associated with aging, offering innovative remedies to enhance quality of life and foster autonomy within the older adult populace [11].

Recent reports have begun to focus on the application of science and technology in the management of older adult health. Chan et al [12] discussed the use of home health monitoring systems in older adult health management through a scoping review. Hong et al [13] provided an overview of the development of smart home technologies for older adults through bibliometric and scientometric analyses. Yenişehir et al [14] analyzed AI technologies for preventing falls among older adults. While these reports demonstrated the potential of technology applications across various aspects, they often focus on specific technologies or address specific health areas for older adults. However, there remains a lack of research exploring a more comprehensive technological revolution across the broader field of older adult health.

The bibliometric analysis methodology equips us with a robust tool set for exploration within this rapidly evolving field. By quantitatively assessing published literature, this method effectively delineates the research hot spots and elucidates the knowledge structures that define scientific activities. It enables us to trace the developmental trajectory and pivotal transformations of digital health and the technological revolution in addressing the health challenges of aging. Notably, over the past 25 years, the integration of technology in older adult health care has seen extensive growth and diversification, with numerous innovative solutions being introduced [15]. This paper aims to comprehensively review the development trends of technology in aging health management and care during this period and look forward to future development prospects. By systematically analyzing the evolution paths of technological solutions, existing achievements, and challenges faced, we will provide a comprehensive understanding of technology applications in the health of older adults. We will also explore possible future development directions to provide important reference and inspiration for promoting the further development of the field of health technology for older adults.

## Methods

### Data Collection

Web of Science stands as the foremost research platform encompassing hard sciences, social sciences, arts, and humanities information [16,17]. Additionally, it serves as the authoritative global citation database, collaborating with the world’s most reputable publishers. We searched the Web of Science Core Collection and selected the Science Citation Index Expanded and Social Sciences Citation Index databases. The search strategy was meticulously designed to encompass a comprehensive array of terms associated with aging, health challenges specific to older adults, and technological interventions that address these issues. The search formula is shown in Textbox 1.

The search period ranged from January 1, 1999, to March 18, 2024; this period was chosen to capture the developments in the field over the last 25 years. The inclusion criteria were strictly articles published in English, focusing on original research and reviews, to ensure the quality and relevance of the data extracted.

**Search formula.**

(TS=(“aging” OR “ageing” OR “elderly” OR “seniors” OR “older adults” OR “gerontology”) AND TS=(“health problems” OR “health issues” OR “healthcare challenges” OR “geriatric health” OR “chronic diseases” OR “mobility issues” OR “cognitive declines” OR “mental health issues” OR “physical health problems” OR “dementia” OR “Alzheimer’s” OR “osteoporosis” OR “arthritis” OR “cardiovascular diseases” OR “diabetes” OR “falls” OR “hearing loss” OR “vision impairment” OR “loneliness” OR “social isolation” OR “well-being” OR “quality of life”) AND TS=(“technological solutions” OR “technology responses” OR “digital health” OR “health technologies” OR “e-health” OR “m-health” OR “telehealth” OR “telemedicine” OR “wearable technologies” OR “assistive technologies” OR “smart healthcare solutions” OR “AI in healthcare” OR “information technology in healthcare” OR “robotic assistance” OR “virtual reality in healthcare” OR “augmented reality in healthcare” OR “IoT in healthcare” OR “internet of things in healthcare” OR “machine learning in healthcare” OR “data analytics in healthcare” OR “blockchain in healthcare” OR “genomic medicine” OR “personalized medicine” OR “remote patient monitoring” OR “predictive analytics” OR “health informatics” OR “biotechnology” OR “nanotechnology”)).

### Data Analysis

Microsoft Office Excel 2019, VOSviewer (v.1.6.20), and CiteSpace (v.6.3.R1 Advanced) were used to analyze all 1172 files. VOSviewer, a bibliometric software, was developed in 2009 by van Eck and Waltman [18] at Leiden University’s Center for Science and Technology Studies in the Netherlands. This Java-based software is free to use and is highly proficient in visualizing and analyzing large-scale data [18]. CiteSpace, created by Chen [19], is a bibliometric and visual analysis tool used for delving into collaborations, priorities, internal structures, focal areas, and potential trends within distinct domains.

## Results

### Annual Scientific Production

**Figure 1:**
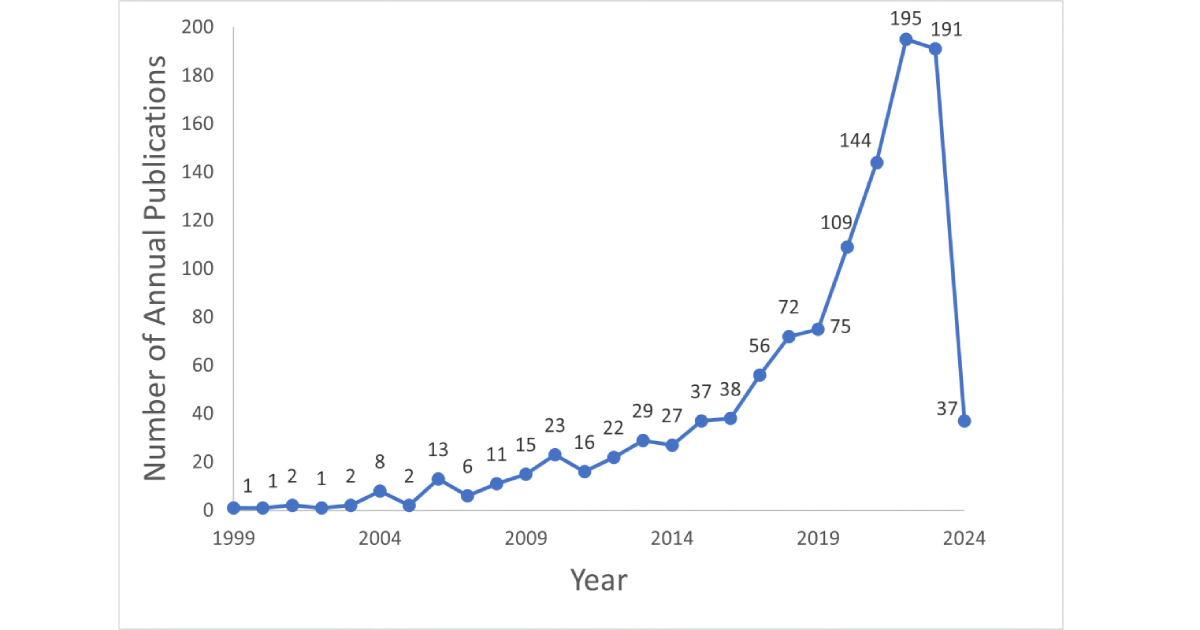
Annual scientific production in technology-based solutions for health challenges in aging.

Our search strategy uncovered 1172 publications from 1999 to 2024, with 1133 being research or review articles, including 827 (73%) research articles and 306 (27%) review articles. The publication trend over the last 25 years showed a gradual but fluctuating increase, starting with single-digit publications in 1999 to a peak of 195 publications in 2022. This upward trajectory experienced a slight decline to 191 publications in 2023. The detailed evolution of these publication numbers, capturing both the growth and the fluctuations, is comprehensively documented in Figure 1.

### National and Institutional Contributions to Scientific Research and Collaborative Endeavors

**Figure 2:**
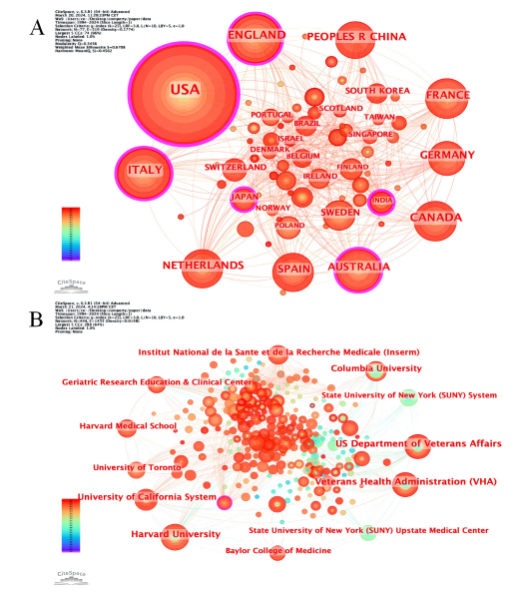
(A) National and (B) institutional contributions to scientific research and collaborative endeavors.

We describe the scientific contributions of each country to research on technological responses to health issues in aging (Figure 2 and Table 1). The top 10 countries most interested in technological solutions to aging health issues, based on the count of publications and their centrality in the research network, are the United States (404 publications, centrality of 0.20), Italy (112 publications, centrality of 0.13), England (102 publications, centrality of 0.13), Canada (91 publications, centrality of 0.06), Australia (81 publications, centrality of 0.11), France (81 publications, centrality of 0.07), the Netherlands (74 publications, centrality of 0.06), Spain (74 publications, centrality of 0.07), People’s Republic of China (72 publications, centrality of 0.05), and Germany (70 publications, centrality of 0.03). The United States, Italy, India, Japan, and Australia have a prominent centrality in the network, a distinction visually emphasized by pink circles in Figure 2. These circles not only highlight the critical roles these countries play but also underscore their extensive interconnections with other nations. This analysis highlights the leading roles these countries play in advancing research focused on addressing health challenges associated with aging through technological innovations.

**Table 1:** National and institutional contributions to scientific research and collaborative endeavors.

Country or institution			Rank		Publications, n		Centrality
**Country**							
	United States	1		404		0.20	
	Italy	2		112		0.13	
	England	3		102		0.13	
	Canada	4		91		0.06	
	Australia	5		81		0.11	
	France	6		81		0.07	
	The Netherlands	7		74		0.06	
	Spain	8		74		0.07	
	People’s Republic of China	9		72		0.05	
	Germany	10		70		0.03	
**Institution**							
	Harvard University	1		44		0.08	
	US Department of Veterans Affairs	2		41		0.04	
	Veterans Health Administration (VHA)	3		41		0.04	
	University of California System	4		33		0.09	
	Columbia University	5		31		0.06	
	Institut National de la Sante et de la Recherche Medicale (INSERM)	6		25		0.04	
	Harvard Medical School	7		24		0.01	
	Geriatric Research Education & Clinical Center	8		23		0.09	
	University of Toronto	9		22		0.02	
	State University of New York (SUNY) System	10		20		0.01	

We also present an analysis of the scientific contributions of various institutions to research on technological responses to health issues in aging (Figure 2 and Table 1). Among the top institutions demonstrating notable interest in technological solutions for aging-related health issues, Harvard University stands out with 44 publications and a centrality of 0.08. Following closely are the US Department of Veterans Affairs and Veterans Health Administration, each with 41 publications and a centrality of 0.04. The University of California System has contributed 33 publications with a centrality of 0.09, while Columbia University follows with 31 publications and a centrality of 0.06. Notably, *Institut National de la Sante et de la Recherche Medicale* has produced 25 publications, maintaining a centrality of 0.04. Harvard Medical School and the Geriatric Research Education & Clinical Center have 24 and 23 publications, respectively, with centrality values of 0.01 and 0.09. Additionally, the University of Toronto has contributed 22 publications with a centrality of 0.02, whereas the State University of New York System has produced 20 publications with a centrality of 0.01.

### Publications Analysis Based on Top Cited Articles

In the retrieved collection, a total of 1133 papers were cited 52,704 times. The top 10 most cited papers, detailed in Table 2, showcase significant contributions to the field. The most cited article, “Lack of exercise is a major cause of chronic diseases” [20], published in *Comprehensive Physiology* in 2012, received 1405 citations. This highlights the critical impact of physical inactivity on health. Among the top 20 most cited papers, journals such as *Comprehensive Physiology*, *Computer Methods and Programs in Biomedicine*, and *IEEE Transactions on Biomedical Engineering* have each made notable contributions, reflecting the interdisciplinary nature of research impacting health and technology. The diversity in topics among these highly cited papers reflects the evolving landscape of health-related research, addressing contemporary challenges such as the COVID-19 pandemic and the aging population.

**Table 2:** The top 10 most cited papers.

Title	Citations, n	Journal	Year
Lack of exercise is a major cause of chronic diseases [20]	1405	*Comprehensive Physiology*	2012
A review of smart homes- present state and future challenges [21]	565	*Computer Methods and Programs in Biomedicine*	2008
Unobtrusive sensing and wearable devices for health informatics [22]	502	*IEEE Transactions on Biomedical Engineering*	2014
Mobile-health: a review of current state in 2015 [23]	489	*Journal of Biomedical Informatics*	2015
Impact of social isolation due to COVID-19 on health in older people: mental and physical effects and recommendations [24]	446	*Journal of Nutrition Health & Aging*	2020
The digital divide among low-income homebound older adults: internet use patterns, eHealth literacy, and attitudes toward computer/internet use [25]	426	*Journal of Medical Internet Research*	2013
Effectiveness of remote patient monitoring after discharge of hospitalized patients with heart failure: the Better Effectiveness After Transition -- Heart Failure (BEAT-HF) randomized clinical trial [26]	412	*JAMA Internal Medicine*	2016
Age-related diseases and clinical and public health implications for the 85 years old and over population [27]	399	*Frontiers in Public Health*	2017
Older adults’ attitudes towards and perceptions of ‘smart home’ technologies: a pilot study [28]	396	*Medical Informatics and the Internet in Medicine*	2004
Smart homes and home health monitoring technologies for older adults: a systematic review [29]	336	*International Journal of Medical Informatics*	2016

### Publications Analysis Based on Authors

In the landscape of this research field, a comprehensive analysis reveals that 1173 papers have been published with contributions from 6086 authors, detailed in Table 3. Among these contributors, Ruth S Weinstock and Steven Shea stand out with 16 publications each, followed by Joseph P Eimicke with 9 publications. These figures highlight not only the prolific nature of certain researchers but also the depth of collaboration and interdisciplinary engagement within the community. The citation metrics further illuminate the impact of these contributions, with Ruth S Weinstock’s works receiving 613 citations, indicating a significant influence within the academic sphere.

Delving deeper into the data, other notable contributors such as Roberto Bernabei, with 6 publications garnering 394 citations, demonstrate the high quality and relevance of research in this area. This detailed examination of publications and citations reveals a vibrant network of scholarly communication, characterized by a rich tapestry of collaboration and intellectual exchange. The distribution of citations, with individuals like Roberto Bernabei achieving an average of 65.67 citations per document, underscores the substantial reach and engagement of the research outputs in this field, which is documented in detail in Table 3.

**Table 3:** Publications analysis based on authors.

Author	Documents, n	Citations, n	Citations per document
Bijan Najafi	16	266	16.63
Steven Shea	16	603	37.69
Ruth S Weinstock	16	613	38.31
Walter Palmas	10	423	42.30
Joseph P Eimicke	9	474	52.67
Rebeca Izquierdo	9	408	45.33
Jeanne A Teresi	8	405	50.63
Roberto Bernabei	6	394	65.67
Philip C Morin	6	159	26.50
Anne Tiedemann	6	19	3.17
Paula M Trief	6	292	48.67

### Keyword Analysis

**Figure 3:**
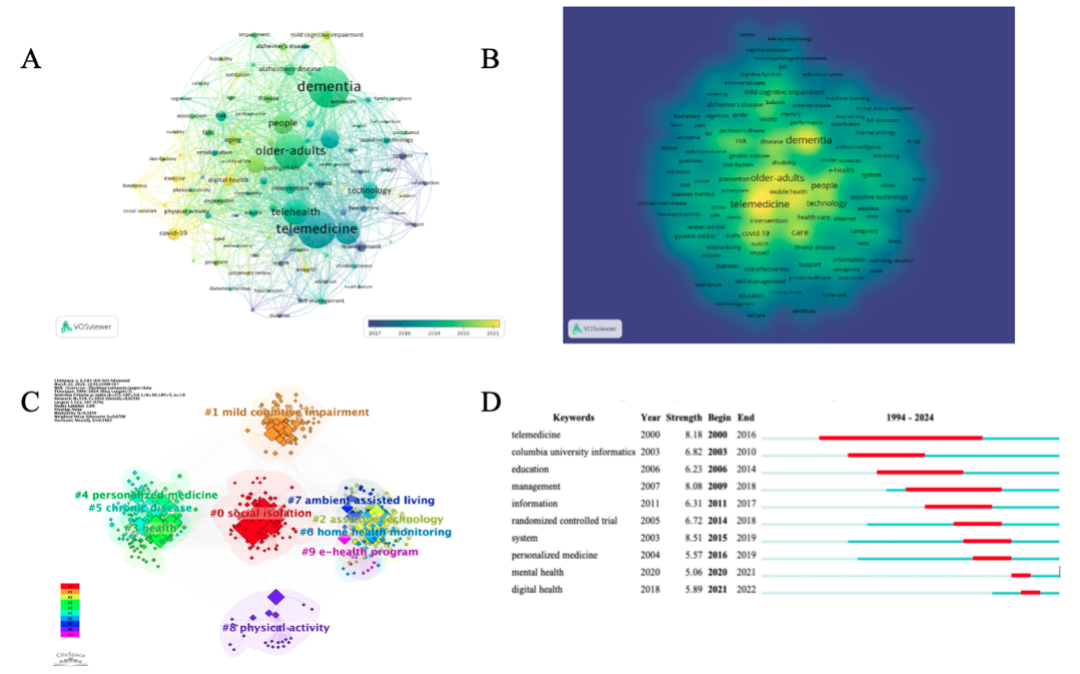
Keyword analysis. (A) Keywords appearing more than 5 times as shown by overlay visualization. (B) Keywords appearing more than 5 times as shown by density visualization. (C) Keyword cluster analysis. (D) The strongest citation bursts of the top 10 keywords.

In this comprehensive analysis, keywords were meticulously selected from the titles and content of research papers, establishing a set of standardized terms that succinctly capture the core themes of the papers’ subject matter. Through the application of VOSviewer, a total of 4690 keywords were identified. Notably, 188 of these keywords appeared more than 10 times, and 102 keywords were mentioned over 20 times. A thorough visual analysis was performed on the top 100 keywords by frequency, offering insights into the most prevalent topics of discussion and research. The analysis was further refined to keywords appearing more than 5 times in Figure 3 to improve clarity and accessibility. The visual mapping of these keywords illustrates the connections between them, with the thickness of the lines between nodes indicating the strength of their associations. This visualization underscores the frequency with which certain keywords co-occur within the same literature, revealing patterns of thematic relevance and interdisciplinary connections.

The predominant keywords identified through this analysis included “dementia,” “telemedicine,” “older-adults,” “telehealth,” “care,” “people,” “health,” “older adults,” “technology,” “covid-19,” “alzheimer’s-disease,” “aging,” “digital health,” “intervention,” and “risk.” These terms not only highlight the core subjects of the analyzed literature but also reflect significant trends and areas of interest within the research community. The emphasis on terms such as “dementia,” “telemedicine,” and “covid-19” points to a strong focus on health care challenges, technological advancements in medical care, and the impact of global health crises. Similarly, the frequent mention of “older adults” and “aging” underscores the growing attention to geriatric care and the health issues associated with aging populations.

Keyword clustering reveals the underlying structure of knowledge within a particular field of research, helping to categorize and define its boundaries. The clusters obtained after analysis mainly included “social isolation,” “mild cognitive impairment,” “assistive technology,” “health,” “personalized medicine,” “chronic disease,” “home health monitoring,” “ambient assisted living,” “physical activity,” and “e-health program.”

Finally, we statistically analyzed the strongest citation bursts of the top 10 keywords (Figure 3). The blue line represents the time interval, and the red line represents the time period when the keyword outbreak was found [30]. The first keyword with citation bursts initially surfaced in 2000 (telemedicine) and remained the most influential keyword until 2016. Conversely, the most recent keyword with citation bursts emerged in 2021 (digital health) and maintain its significance until 2022.

## Discussion

### Principal Findings

In this research, we used bibliometric analysis techniques for a comprehensive examination of literatures related to the topic of smart health care published prior to March 18, 2024. Our analysis shows that the field of technology-based solutions for health challenges in aging is gradually becoming the focus of widespread attention. In the past 5 years, there has been a sharp increasing trend. Notably, the prevalence of research-oriented literatures significantly outweighs that of reviews. This illustrates the complexity of the field that needs to be addressed, as the research literature often provides original research results and new insights.

Among the top 10 publishing countries, comprising 2 from the Americas, 6 from Europe, 1 from Asia, and 1 from Oceania, their collective output constitutes 99% of the total articles. These countries not only boast high gross domestic products but also house diverse aging populations, providing a rich backdrop for research into older adult health technology. For example, the United States and Italy not only possess robust economies but are also confronted with profound aging-related challenges. This compels them to prioritize and allocate significant funding to research initiatives aimed at mitigating these issues. Similarly, England, Canada, and Australia leverage their economic strengths with strategic investments in health technology, effectively addressing the needs of their expanding older adult populations. Furthermore, the central role these countries play in international research collaborations underlines their influence in shaping global research agendas. Their economic capacity enables higher research capabilities and outputs, while the extensive aging of their populations demands innovative solutions to increasingly complex health challenges. On the other hand, emerging economies such as China, despite their lower overall research outputs, are experiencing rapid growth in this area. This is propelled by urgent demographic shifts and an increasing governmental focus on health care innovation [31]. This diverse international landscape showcases the pivotal role of economic and demographic factors in driving the direction and intensity of research into aging and health technology.

Keywords serve as indicators of the central themes and primary content within a paper, thus offering a concise representation of the research’s focal points. Our use of cluster analysis based on these keywords has enabled the identification of 10 distinct categories. Furthermore, by analyzing the occurrence of explosive words, we gain deeper insights into the intricate nuances and broad spectrum of the older adult health technology field. The current research hot spots focus on older adults with cognitive impairment, social isolation, and chronic diseases. Interventions include assistive technology, personalized medical solutions and eHealth projects, implementing remote consultation, education, monitoring, triage, care, and health management for older adults.

Social isolation can lead to an increase in mental and physical health problems, which is an important health challenge for older adults, especially those with cognitive conditions, so taking steps to reduce social isolation is critical to the health of older adults [32,33]. Assistive technology plays an important role in this regard. For example, ambient assisted living technology can provide a convenient and safe home environment and reduce the burden of life for older adults [34]. Virtual reality technology and computer-aided technology can detect very subtle cognitive impairment, help diagnose earlier, and prevent or delay the occurrence of dementia [35]. In addition, personalized medical solutions and eHealth projects through computer, iPad, or smartphone applications can also provide customized medical services to older adults to meet their individual health needs, thereby improving their quality of life [36-39].

On the other hand, older adults often face challenges with chronic conditions that require ongoing health monitoring and management [40]. Home health monitoring and telemedicine solutions provide older adults with convenient health management methods and help timely intervention and treatment of chronic diseases [41]. Additionally, by encouraging older adults to participate in physical activity and providing remote guidance and monitoring, their physical and mental health can be improved, thereby reducing the risk of chronic disease [42,43]. To sum up, by combining various means such as assistive technology, personalized medicine, health monitoring, and education, the health level of older adults can be comprehensively improved and the process of healthy aging can be promoted.

The analysis results show that the focus of the research shifted from “telemedicine” to “digital health.” Digital health emphasizes more on the health and well-being of individuals and populations rather the diseases and patients [44]; has a broad scope; and includes the use of wearable devices, mobile health, telehealth, health information technology, and telemedicine [45]. This paradigm shift reflects an evolving health care approach where technology extends beyond remote clinical interactions to a comprehensive system that enhances personalized health management. Digital health integrates a variety of innovative technologies that facilitate not only remote monitoring but also proactive health management strategies. These tools empower patients through real-time data, engaging them in their own health management and improving the responsiveness of health care systems to their needs.

Indeed, the technological revolution has played a significant role in addressing key areas such as chronic disease management, mobility support, social connectivity, and cognitive enhancement. Chronic disease management has seen innovative applications of AI and machine learning to predict and manage episodes in conditions like diabetes and heart disease, reducing hospital visits and improving quality of life [46,47]. Mobility impairments, often a barrier to independent living, are being tackled with advanced robotics and smart wearables that assist movement and monitor falls [48,49]. Social isolation, exacerbated by physical distancing during the COVID-19 pandemic, is being mitigated through virtual reality platforms that allow social interactions and community building without physical presence [50]. Furthermore, cognitive decline—one of the most challenging aspects of aging—is being tackled with cognitive training apps and platforms that stimulate brain activity and decelerate the progression of diseases like Alzheimer disease [51]. These technology-driven innovations underscore the transformative role of digital health in enhancing health management for older adults.

Our research findings reveal a significant shift toward a more integrated and personalized approach in older adult health management through the application of digital health technologies. This shift from traditional telemedicine to a comprehensive digital health system emphasizes a greater focus on enhancing individual health outcomes and overall well-being, rather than solely treating diseases. Our analysis underscores the transformative role of technology in health care, particularly through innovations such as AI, smart wearables, virtual reality, and ambient assisted living technologies, which collectively improve the quality of life and independence of the older adult population. Future research should aim to address the barriers to adoption and implement strategies that ensure these technological solutions are accessible, equitable, and tailored to the diverse needs of aging populations worldwide. Additionally, further studies are needed to evaluate the long-term impacts of these technologies on health outcomes and to understand better how they can be effectively integrated into standard care practices. This will not only help in advancing the field of digital health but also in shaping policies and practices that support healthy aging in various socioeconomic contexts.

While this study provides valuable insights, it is imperative to acknowledge its limitations. Primarily, our analysis draws upon data solely from the Science Citation Index Expanded and Social Sciences Citation Index databases, thus excluding studies from non–Sciences Citation Index journals, alternative databases, or conference presentations from our assessment. Additionally, it is essential to recognize the temporal influence on citations, whereby newly published works may receive fewer citations compared to earlier ones over time.

### Conclusion

With the increasing trend of global population aging, technology-based solutions in older adults have developed rapidly, especially in the past decade. In the past 25 years, the focuses of the research are on older adults with chronic diseases, cognitive impairment, and social isolation, and some progress has been made in remote monitoring, education, personalized medicine, care, and ambient assisted living. The recent research focus has shifted toward digital health, indicating health and prevention. More research can be conducted in areas such as prediction, prevention, health management, and appropriate technological innovation in the future. At the same time, international cooperation should continue to be strengthened. Resource-limited countries can learn from the methods and strategies of resource-rich countries, combine their own characteristics, and provide more support for older adults.

## Abbreviations

AI: artificial intelligence
